# A Bimodal Fluorescence-Raman Probe for Cellular Imaging

**DOI:** 10.3390/cells10071699

**Published:** 2021-07-05

**Authors:** Jiarun Lin, Marcus E. Graziotto, Peter A. Lay, Elizabeth J. New

**Affiliations:** 1 School of Chemistry, The University of Sydney, Sydney, NSW 2006, Australia; jiarun.lin@sydney.edu.au (J.L.); marcus.graziotto@sydney.edu.au (M.E.G.); 2 The University of Sydney Nano Institute (Sydney Nano), The University of Sydney, Sydney, NSW 2006, Australia; 3 Sydney Analytical, The University of Sydney, Sydney, NSW 2006, Australia; 4 Australian Research Council Centre of Excellence for Innovations in Peptide and Protein Science, The University of Sydney, Sydney, NSW 2006, Australia

**Keywords:** fluorescent probe, Raman spectroscopy, multimodal imaging, lipid droplets

## Abstract

Biochemical changes in specific organelles underpin cellular function, and studying these changes is crucial to understand health and disease. Fluorescent probes have become important biosensing and imaging tools as they can be targeted to specific organelles and can detect changes in their chemical environment. However, the sensing capacity of fluorescent probes is highly specific and is often limited to a single analyte of interest. A novel approach to imaging organelles is to combine fluorescent sensors with vibrational spectroscopic imaging techniques; the latter provides a comprehensive map of the relative biochemical distributions throughout the cell to gain a more complete picture of the biochemistry of organelles. We have developed **NpCN1**, a bimodal fluorescence-Raman probe targeted to the lipid droplets, incorporating a nitrile as a Raman tag. **NpCN1** was successfully used to image lipid droplets in 3T3-L1 cells in both fluorescence and Raman modalities, reporting on the chemical composition and distribution of the lipid droplets in the cells.

## 1. Introduction

Understanding the biochemical composition of cells and organelles is essential for understanding both physiological and pathological processes. The comprehension of the chemistry of cells is greatly aided by tools to both identify and monitor the chemistry of organelles in cellulo. Small molecule fluorescent probes have emerged as important tools for investigating cells. Most advantageously, fluorescent probes can respond to specific biochemical species or stain specific sites on the cell, while being imaged at a high spatial and temporal resolution [1]. However, most fluorescent sensors can only target one or two analytes of interest and crosstalk can complicate the use of multiple fluorophores to investigate different species. Fluorescent probes are less useful for investigating the general chemical composition of the cell and must be combined with other techniques to gain a more complete picture of the biochemical environment.

Raman spectroscopy is a label-free and non-destructive technique that is increasingly being used to characterise biological specimens [2,3,4,5]. It is reliant on the inelastic scattering of monochromatic light upon interactions with molecular vibrations in a sample, which shifts the energy of the light based on the functional groups present [3]. Certain spectral bands are representative of classes of biomolecules, and the distribution of multiple bands (biochemical classes) can be mapped simultaneously throughout a sample [6].

Multimodal imaging is gaining traction in biomedical and clinical studies as it combines the relative advantages of two or more imaging techniques [7,8]. In general, multimodal imaging that provides a precise colocalization of fluorescence microscopy images with other modalities requires the development of a fluorescent probe molecule that can simultaneously or sequentially report on each modality [9,10,11]. Such systems are excellent examples of chemistry aiding in the elucidation of biological systems. Bimodal fluorescence and Raman techniques can be used to provide complementary information on the cellular environment. As fluorescence is a competing physical phenomenon to inelastic scattering, fluorescence background can be a significant issue in Raman spectroscopy, though this issue can be overcome with the use of near-infrared excitation and background subtraction [12]. Several approaches for multimodal fluorescence and Raman imaging have been investigated. The combination of tissue autofluorescence and Raman spectroscopy has been used in diagnostics to provide a more accurate result of the biomolecule distribution than either modality alone [13,14,15]. Fluorescent probes have also been used to support the conclusions of separately performed Raman mapping, or to select areas of interest in large samples for Raman studies [16]. Nanoparticle-based fluorophores have been used in surface-enhanced Raman scattering (SERS) studies [17,18,19,20,21]; however, these are relatively complex systems in terms of synthesis and have a large size and weight compared to most biomolecules that are more likely to induce biochemical changes to the surrounding intracellular environment.

In recent times, the use of bioorthogonal Raman tags, such as alkyne and nitrile groups, or C≡O ligands in fluorescent metal complexes, with signals in the biologically silent region of the spectrum from 1800 to 2800 cm^−1^, has offered a new way of tracking the uptake of small molecules [9,22,23,24,25]. The development of molecular probes incorporating a bioorthogonal Raman tag and a fluorophore provide complementary information on localisation and the biochemical environment. Despite this promise, few examples of such probes have been reported to date. Li et al. reported a dual aggregated induced emission fluorescence and (AIE) stimulated Raman scattering mitochondrial probe, AIE-SRS-mito, incorporating both an alkyne and a nitrile group [26]. de Pablo et al. reported a small molecule fluorescent photosensitiser incorporating an alkyne, DC473, as a photodynamic therapy agent that could be imaged with both modes [27]. Considering the advantages of both techniques, the development of multimodal probes from this approach provides orthogonal information regarding the biodistribution of molecules throughout the cell.

Lipid droplets are the major organelles for lipid storage and are found in virtually every cell type [28]. They consist of a core of neutral lipids, mostly triglycerides and sterol esters, surrounded by a phospholipid monolayer [29,30]. While once considered inert storage bodies in the cytoplasm, lipid droplets are now known to dynamically interact with all major organelles [31]. It is thus beneficial to develop probes that can examine the chemical environment of lipid droplets. While there are a few commercially available fluorescent probes for lipid droplets, most stain the entire droplet without reporting on the chemical composition. A vibrational spectroscopy analysis of lipids can be complicated by spectral overlap. Bader et al. reported ReZolve-L1™, a tricarbonyl rhenium-based probe for polar lipids that was used in bimodal fluorescence-Raman studies [9,10]. We sought to develop a lipid droplet directed small molecule fluorophore with a Raman tag to allow for specific tracking of the probe in both modalities.

## 2. Materials and Methods

### 2.1. Photophysical Studies

The solvents used in the photophysical studies were dichloromethane (HPLC grade, Sigma-Aldrich, Sydney, Australia), acetonitrile (Spectroscopy Grade, AJAX, Themo Fisher Scientific, Melbourne, Australia), absolute ethanol (200 proof, HPLC/spectrophotometric grade, Sigma-Aldrich, Sydney, Australia), or HEPES (20 mM, pH 7.4; Combi-Blocks Inc., San Diego, CA, USA). All compounds were prepared as stock solutions in DMSO (Spectroscopy grade, Sigma-Aldrich, Sydney, Australia) and the concentrated stock solution was diluted to the required concentration in the appropriate solvent. The DMSO concentration in all experiments was <0.5% *v*/*v*.

Absorption spectra were obtained for each compound in absolute ethanol on a Varian Cary 400 UV-Vis spectrophotometer (Agilent, Melbourne, Australia) using 10 mm pathlength quartz cuvettes. Fluorescence spectra were obtained for each compound in dichloromethane, acetonitrile, absolute ethanol, or a HEPES buffer on a Varian Cary Eclipse fluorometer using 10 mm pathlength quartz cuvettes.

### 2.2. Preparation of Solutions for Cell Culture

For the differentiation of 3T3-L1 cells, stock solutions of differentiation reagents were prepared. Insulin (Sigma-Aldrich, Sydney, Australia) was prepared as a 2 mg/mL solution in 0.01 M aqueous hydrochloric acid (AJAX, Themo Fisher Scientific, Melbourne, Australia) and syringe-filtered. Dexamethasone (Sigma-Aldrich, Sydney, Australia) was prepared as a freezer stock solution of 10 mM stock solution in chromatography grade ethanol (Merck Pty Ltd., Melbourne, Australia), and was further diluted to a working stock solution of 1 mM in phosphate buffered saline, pH 7.4 (PBS; Thermo Fisher Scientific, Melbourne, Australia). 3-*Iso*-butyl-1-methylxanthine (IBMX; Sigma-Aldrich) was prepared as a 50 mM stock solution in dimethyl sulfoxide (DMSO; Sigma-Aldrich).

Ammonium acetate solution (0.1 M) was prepared via the dissolution of ammonium acetate (Merck Pty Ltd., Melbourne, Australia) in Milli-Q® ultrapure deionised water (Merck Millipore, Melbourne, Australia). The solution was syringe-filtered prior to use.

### 2.3. Cell Culture

All cell lines were purchased from the American Type Culture Collection (Manassas, VA, USA). All cell lines were maintained at 37 °C in 5% carbon dioxide.

3T3-L1 murine pre-adipocyte cells were subcultured in a maintenance medium consisting of Dulbecco’s modified Eagle’s medium (DMEM; Thermo Fisher Scientific, Melbourne, Australia) supplemented with 10% FBS, 1% Gibco^®^ GlutaMAX™ Supplement (Thermo Fisher Scientific, Melbourne, Australia), 100 units/mL penicillin, and 100 µg/mL streptomycin (Thermo Fisher Scientific, Melbourne, Australia). The passage number was kept below 10 and confluency was not allowed to exceed 80%.

To differentiate pre-adicocytes from mature adipocytes, 3T3-L1 pre-adipocyte cells were plated at a confluency of approximately 7500 cells/cm^2^. After 2 d, the medium was replaced with fresh maintenance media. After a further 2 d, the medium was replaced with maintenance media freshly supplemented with 1 µg/mL insulin, 1 µM dexamethasone, and 0.5 mM IBMX (day 0 post-differentiation). After another 3 d, the cells were washed with PBS and the medium was replaced with maintenance media freshly supplemented with 1 µg/mL insulin (day 3 post-differentiation). Then, after 2 d, the medium was replaced every 2–3 d with fresh maintenance media until day 14 post-differentiation, where they were used in endpoint experiments.

### 2.4. Dosing for Imaging Experiments

Nile Red (Sigma-Aldrich) and **NpCN1** (synthesized as described in the ESI) were prepared as concentrated stock solutions in DMSO. Cells were treated with either 20 μM of **NpCN1** or 10 μM Nile Red in a fresh maintenance medium, or the equivalent volume of DMSO, with the final DMSO concentration < 1% in all cases. The cells were then incubated at 37 °C in 5% carbon dioxide atmosphere for 4 h.

After treatment, the cells were washed with warm PBS (3 × 0.5 mL) and then imaged in FluoroBrite DMEM media (FDMEM, Thermo Fisher Scientific, Melbourne, Australia) supplemented with 10% FBS and 1% Gibco® GlutaMAX™ Supplement (Thermo Fisher Scientific, Melbourne, Australia).

### 2.5. Live Confocal Microscopy

3T3-L1 pre-adipocyte cells were seeded in poly-D-lysine coated 3.5 mM glass bottom dishes (MatTek Corporation, Ashland, MA, USA) and differentiated as described above before dosing.

Cells were dosed as described above before imaging. Images were obtained at 37 °C in 5% carbon dioxide atmosphere on an Olympus FluoView FV3000 Confocal Laser Scanning Microscope (Olympus, Melbourne, Australia), equipped with an Olympus 60X water objective (UPLSAPO60XW) and 405, 488, and 561 nm lasers. Emission was collected for each of the lasers from 450 to 550 nm, 500–600 nm, and 570–670 nm, respectively.

Images were processed using FIJI software [32].

### 2.6. Preparation of Fixed Cells on Calcium Fluoride

For upright confocal microscopy and Raman studies, calcium fluoride windows (Crystran Ltd., Poole, UK) were sterilised via soaking in 80% ethanol. Slides were then sequentially dipped in 80% ethanol, PBS, and a maintenance medium before immediate transfer to the dry surface of a 24-well plastic cell culture plate (Corning, Melbourne, Australia). The 3T3-L1 pre-adipocyte cells were then seeded and differentiated as described above.

Cells were then dosed and incubated with 20 μM of **NpCN1** or an equivalent volume of DMSO for 4 h, as described above. After incubation, the windows were washed three times with warm PBS, then once with 0.1 M ammonium acetate solution. The slides were then fixed by rapid immersion in ice-cold methanol (253 K) and then air-dried before use [33].

### 2.7. Upright Confocal Microscopy

Images of cells on CaF_2_ slides were obtained with a Nikon C2 Basic Confocal Microscope (Nikon Australia PTY LTD, Sydney, Australia), equipped with a CFI Plan Apo λ 40X air objective and a 488 nm laser. Emission was collected from 500 to 550 nm.

### 2.8. Raman Spectroscopy

All Raman spectroscopy was conducted with a Renishaw Raman InVia Qontor Microscope (Renishaw plc., Wotton-under-Edge, UK). The instrument was attached to an air-cooled charge-coupled cooling device (CCD), and equipped with edge filters and two gratings (1200 mm/line (visible) and 2400 mm/line (NIR)). The Raman microscope was used with a 50×/0.75 NA objective and a video camera that allowed for direct sample viewing. The software Renishaw WiRE™ (Version 5.3, Renishaw plc., Wotton-under-Edge, UK; WiRE 5.3) was used to control the instrument via a PC computer.

An internal wavenumber calibration was performed before data collection using an internal silicon standard. For Raman mapping, samples were then excited using a XTRA NIR laser (TOPTICA Photonics AG, Graefelfing, Germany) emitting at 785 nm. Spectra were not corrected for instrument response.

For solid **NpCN1**, the spectrum was recorded using the ×50/0.75 NA objective over 100–3200 cm^−1^ with the sample exposed to 1% laser power (~1.5 mW at the sample) for 10 s over three accumulations.

Two-dimensional spectral maps of cell samples on CaF_2_ slides were collected in StreamLine™ mode over the spectral ranges 715–1806 cm^−1^ and 1327–2304 cm^−1^, centred around 1300 cm^−1^ and 1850 cm^−1^, respectively. StreamLine™ is a line-scanning mode and samples were exposed to a laser power of 100% (~150 mW at the sample) for 10 s with a step size of 1.4 μm.

### 2.9. Post-Processing of Raman Spectra

For the spectrum of solid **NpCN1**, background subtraction was performed in WiRE 5.3 using the intelligent polynomial algorithm, with a polynomial order of nine and a noise factor of 1.5. The fitting mode was through regions and intelligent polynomial anchor end points were enabled. The spectrum was then smoothed in the same software using a Savitzky–Golay filter with the parameters of a smooth window of 17 and a polynomial order of 2.

For spectral maps, post-processing was performed on WiRE 5.3 to remove cosmic rays and apply noise filtering. No further processing was performed. The software was then used to generate false colour images using signal-to-baseline for bands of interest. For multivariate analysis, principle component analysis (PCA) maps were generated with Standard Normal Variate (SNV) normalisation.

Representative spectra from maps were exported from WiRE 5.3 and plotted in GraphPad Prism 8 (GraphPad Software, San Diego, CA, USA).

Non-cell regions in each map were identified using the first principal component map generated in WiRE 5.3. This region was then masked out; an average spectrum of the remaining cell spectra was then obtained. To compare cells dosed with **NpCN1** and the vehicle control, these average spectra were exported into GRAMS/AI (.spc) format. An additional PCA was performed on the extracted spectra using The Unscrambler X (v11, CAMO Analytics, AspenTech, Bedford, MA, USA). Spectra were first normalised using the Standard Normal Variate (SNV) method to reduce spectral variability from sampling prior to analysis. A PCA was then performed using the singular value decomposition (SVD) algorithm and cross-validation. Variable weights were all set to 1.0.

## 3. Results

### 3.1. Design and Synthesis of NpCN1

**Figure 1:**
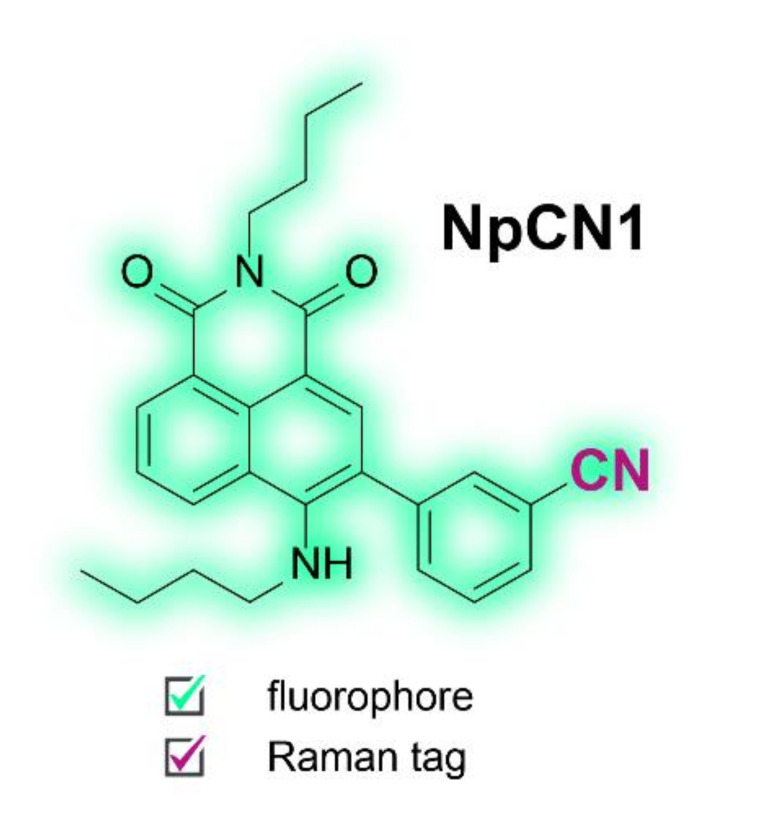
Chemical structure of **NpCN1**, highlighting the dual fluorescent and Raman modalities.

**Figure 2:**
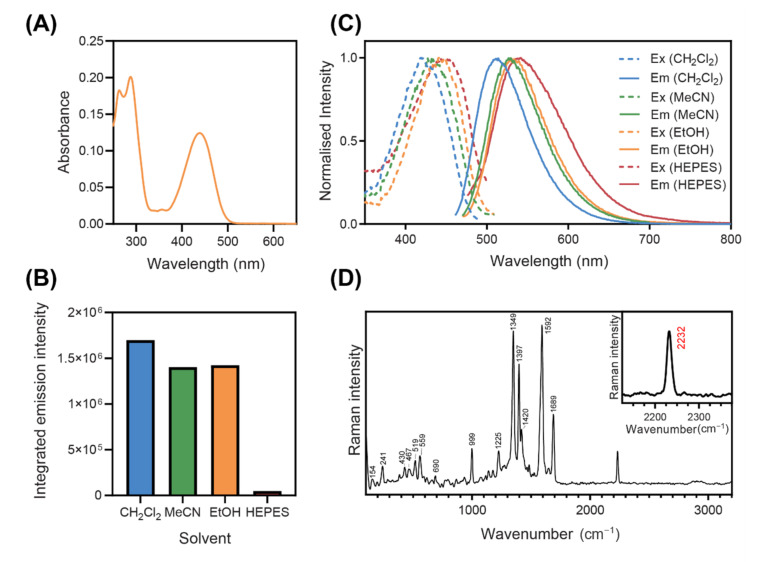
(**A**) Absorbance spectrum of **NpCN1** (10 µM) in absolute ethanol. (**B**) Normalised fluorescence excitation and emission spectra and (**C**) integrated fluorescence emission intensity of **NpCN1** (10 µM) in a variety of solvents; ex = excitation, em = emission, CH_2_Cl_2_ = dichloromethane, MeCN = acetonitrile, EtOH = ethanol. (**D**) Raman spectrum of **NpCN1**, in the solid state, taken with the 50X and 785 nm laser excitation; inset shows the 2232 cm^−1^ C≡N band tracked in this study.

1,8-Naphthalimides are a class of fluorophores commonly used in imaging due to their biocompatibility, chemical stability, photostability, high quantum yields, and large Stokes shifts [34]. Whilst the core structure is easily modified for different applications, naphthalimides have seen little use in fluorescence multimodal imaging probes and are yet to be functionalised for vibrational spectroscopy. The nitrile functional group has been recognised as a suitable Raman tag, but the spectral signature is relatively weak [22]. By attaching it to the naphthalimide core as a benzonitrile, we sought to change the polarizability and increase the intensity of the peak in Raman spectroscopy to create a probe suitable for use in both modalities. For this purpose, we designed and synthesized **NpCN1**, a 4-amino-1,8-naphthalimide with a 3-benzonitrile substituted at the 3-position (Figure 1). We had also tested alkyne tagged naphthalimides [35,36] (Scheme S2, Figure S1), but the benzonitrile tagged naphthalimide had superior properties (Figure 2).

**NpCN1** was synthesised in a three-step reaction (Scheme S1). First, the condensation of *n*-butylamine with 4-bromo-1,8-naphthalic anhydride gave 4-butylamino-*N*-butyl-1,8-naphthalimide [37]. The subsequent bromination of 1 afforded 3-bromo-4-butylamino-*N*-butyl-1,8-naphthalimide, which was subjected to a Suzuki cross-coupling reaction with 3-cyanophenylboronic acid to produce **NpCN1** in good yields (for complete organic synthetic procedures, see ESI).

### 3.2. Photophysical Behaviour and Raman Characterisation

**NpCN1** has two main absorption bands in the UV and blue regions of the spectrum (Figure 2A). The fluorescence emission is highly solvatochromic, with strong blue-green fluorescence in non-polar solvents, such as dichloromethane, and is red-shifted in more polar solvents (Figure 2B). The fluorescence intensity of **NpCN1** is also greater in non-polar solvents compared to polar solvents (Figure 2C), with a small difference from dichloromethane to acetonitrile and ethanol, and a substantial 35-fold decrease in a HEPES buffer. These spectral properties are similar to those of the commercial lipid stain Nile Red, which is also highly fluorescent and blue-shifted in non-polar lipids, red-shifted in more polar lipids, such as phospholipid membranes, and quenched in water [38,39,40]. **NpCN1** thus emerged as a potential stain for lipid droplets and other lipid environments in the cell that may act in a similar manner to Nile Red.

Nile Red does not have peaks in the biologically silent region of the FTIR spectrum [41,42], and is thus a poor candidate for Raman studies. However, Raman spectroscopy of solid **NpCN1** has a nitrile peak at 2232 cm^−1^, characteristic of the stretching vibration of benzonitriles that occurs around 2240–2220 cm^−1^ (Figure 2D) [43]. As this is the only prominent peak within the biologically silent region from 2800 to 1800 cm^−1^, this enabled intracellular Raman spectroscopic mapping. Encouragingly, there was little fluorescence background in the original spectrum without background subtraction (Figure S2). Fluorescence and Raman are competing processes, and fluorescence background can overwhelm the usable Raman signal; this is likely due to the 785 nm laser falling out of the range for both single and two photon excitation of the fluorophore. This made **NpCN1** a good candidate for bimodal cell imaging studies.

### 3.3. Cell Viability and Live Cell Microscopy

After establishing the photophysical properties and characterising the Raman spectrum of **NpCN1**, its subcellular localisation in cultured cells was tested.

3T3-L1 differentiated murine adipocyte cells were chosen for their large lipid droplet size of up to 150 µm [44]. Cells dosed with a 50 µM probe in 24 h cell viability assays (detailed in SI) had a cell viability of 97% compared to undosed cells, comparable to the cell viability of the vehicle control (Figure S3). As these conditions were well above the dose and incubation times used in our studies, it was concluded that **NpCN1** had a minimal impact on the cell viability of 3T3-L1 adipocytes and, thus, we proceeded to imaging studies.

**Figure 3:**
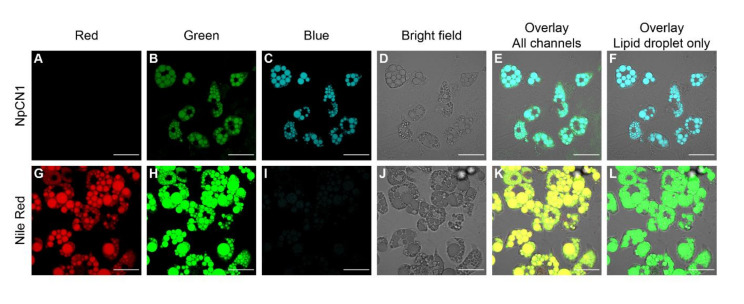
Confocal microscopy images of live 3T3-L1 adipocytes treated with (**A**–**F**) **NpCN1** (20 μM, 4 h) or (**G**–**L**) Nile Red (10 μM, 4 h). Live cells were imaged in (**A**,**G**) red (λ_ex_ = 561 nm, λe_m_ = 570–670 nm), (**B**,**H**) green (λ_ex_ = 488 nm, λe_m_ = 500–600 nm) and (**C**,**I**) blue (λ_ex_ = 405 nm, λ_em_ = 450–550 nm) channels, along with (**D**,**J**) bright field images. (**E**,**K**) shows the overlay of all channels; (**F**) shows the overlay of C and D, (**L**) shows the overlay of H and K. The scale bar on all images represents 50 μm.

Upon laser excitation, 3T3-L1 differentiated adipocytes treated with **NpCN1** showed significant fluorescence in distinct cytoplasmic compartments that appeared to be the lipid droplets that were also visible in brightfield images (Figure 3). In order to confirm this localization, adipocytes were separately stained with Nile Red. While the significant crosstalk between Nile Red and **NpCN1** emission spectra prevented accurate co-staining, the images obtained from staining with each dye are clearly comparable. Both **NpCN1** and Nile Red exhibited solvatochromic properties; in both cases, the more red-shifted channel (Figure 3B,G) showed some non-lipid droplet specific staining evident in the overlay (Figure 3E,K). As Nile Red is known to stain phospholipids in the red channel [45], this is likely to be the intracellular membranes. We observed a similar staining pattern in 3T3-L1 adipocytes treated with a shorter incubation time (Figure S6). **NpCN1** is thus a lipid stain with a similar efficacy and with similar properties as Nile Red in cellulo.

### 3.4. Raman Mapping of 3T3-L1 Cells Dosed with NpCN1

The complementary use of Raman spectroscopy allows for a more complete understanding of **NpCN1**’s localisation in the cell and its association with lipids and other biomolecules. Due to the lower temporal resolution of Raman mapping compared to fluorescence studies, fixation is required to preserve the sample between spectral acquisitions of the maps of different cells. This is also required since live cells change their morphology and biochemical distribution with time and, hence, fixation is required for the colocalisation of spectral features associated with specific cellular substructures. We have previously used the rapid ice-cold methanol method of fixation for 3T3-L1 cells, which allows for minimal damage to the sample [3,10,33].

**Figure 4:**
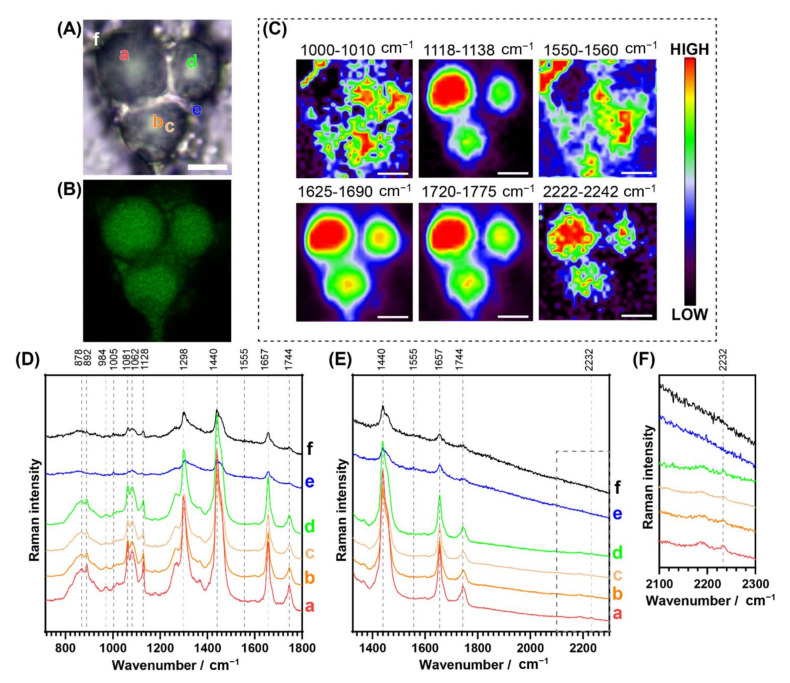
Bimodal cell studies of a representative fixed 3T3-L1 cell dosed with **NpCN1** (20 μM, 4 h). (**A**) shows the bright field and (**B**) shows the confocal fluorescence image of the cell. (**C**) shows the Raman maps with the distribution of phenylalanine (1000–1010 cm^−1^), lipid C-C/fatty acids (1118–1138 cm^−1^), tryptophan (1550–1560 cm^−1^), lipid C=C (1625–1690 cm^−1^), lipid esters (1720–1775 cm^−1^), and **NpCN1** (2222–2242 cm^−1^); maps obtained via calculation of the signal-to-baseline of spectra. (**D**,**E**) show selected spectra extracted from locations **a**–**f** as shown in (**A**), while (**F**) shows detail of dotted region in (**E**). The scale bar represents 10 μm for all images and maps.

Confocal fluorescence microscopy of fixed 3T3-L1 adipocytes incubated with **NpCN1** revealed a similar cellular distribution of the compound as in the live cell studies, where the dye stained the large lipid droplets embedded in the cytoplasm (Figure 4A,B). Line-scan Raman mapping was undertaken on the same cells using 785 nm excitation. False-color maps showed the relative integrated intensity of particular spectral bands at different spatial positions across the cell (Figure 4C). To illustrate the distribution of lipids within the 3T3-L1 cells, the area under the bands centred around 1128 cm^−1^, 1657 cm^−1^, and 1744 cm^−1^ were mapped, corresponding to various lipids (Figure 4C). The band around 1128 cm^−1^ is assigned with the trans-conformation of the **v**(C-C) skeletal backbone in lipids, with some contribution from fatty acids [46]. The band around 1657 cm^−1^ is assigned to the **v**(C=C) mode in aliphatic chains with expected contributions from both polar and non-polar lipids, though there is some overlap with the amide I band of proteins [9,46,47]. The band around 1744 cm^−1^ is assigned to the **v**(C=O) lipid mode, with the main expected contributions from triacylglyciderides (TAGs) and cholesterol esters [9,47]. This same process was repeated for the 2232 cm^−1^ C≡N band of **NpCN1**, which showed a high correlation to the three lipid bands and supports the localisation of the probe in lipid-rich environments. The distribution observed in the bands also overlapped with the localisation present in the fluorescence image, providing a bimodal confirmation of cellular lipid localisation. As expected, the C≡N band at 2232 cm^−1^ of **NpCN1** was absent in the cells dosed with the vehicle control only (Figure S4). The band centred around 1005 cm^−1^, assigned to phenylalanine [46], and 1555 cm^−1^, assigned to tryptophan and amide II [46], have no significant overlap with lipid, carbohydrates, and nucleic acid; these maps were representative of protein distribution throughout the cell. The two maps were largely correlated to each other and were not correlated to **NpCN1**, further confirming localisation.

Raman spectra were extracted from regions within a Raman map of high ***a***, medium ***b***–***d***, and low ***e***–***f*** intensity of the band at 2232 cm^−1^ (Figure 4D,E). While this peak was relatively small, this was due to the low intracellular concentration of the probe compared to the major native biomolecules that make up the cell, but the probe band intensity was well above the Raman signal-to-noise ratio in the regions where the probe was detected. The strongest signal was observed in ***a*** in the core of the droplet with the strongest lipid bands; the signal was not observable in ***e***–***f***, regions that contain high protein and low lipid levels. The maps and spectra confirm that **NpCN1** accumulated in the lipid droplets and did not accumulate in other lipophilic regions of the cell at any appreciable concentrations.

### 3.5. Principal Component Analysis of Raman Spectral Maps

**Figure 5:**
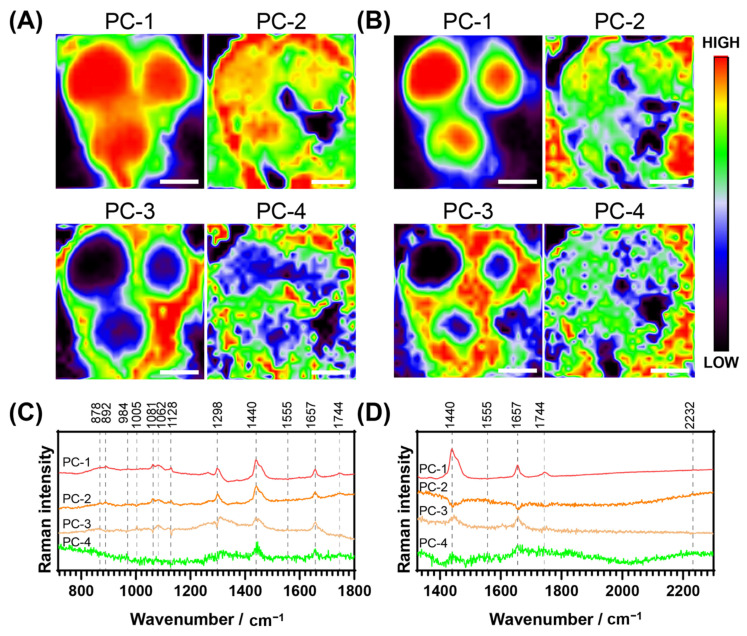
Principal component analysis of the Raman spectra of a cell dosed with **NpCN1** (20 μM, 4 h); same representative cell as shown in Figure 4. (**A**,**B**) show the PC maps of each of the scores of the four main PC of the spectra collected from 715–1806 cm^−1^ and 1327–2304 cm^−1^, respectively. (**C**,**D**) show the loadings of the scores mapped in (**A**,**B**), respectively. The scale bar represents 10 μm for all maps.

Principal component analysis (PCA) is a multivariate analysis technique used to reduce dimensionality in large datasets. Spectroscopic techniques such as Raman mapping often generate thousands of spectra per dataset, so PCA provides an objective assessment to identify patterns and trends that cannot be easily analysed otherwise. A PCA of one of the maps of a representative cell incubated with **NpCN1** is shown in Figure 5A,B, with the loadings of these principal components (PCs) in Figure 5C,D. The PC-1 for both sets of maps corresponds strongly with that of the average cell spectrum, with the cell scoring more highly than the background; however, the thin membrane protrusions defining the boundary of the cell are much better defined than in any of the optical or fluorescence images, or any of the maps based on individual bands (Figure 4). This loadings plot corresponds well with the Raman spectra of triglycerides of the unsaturated fatty acids and oleic and palmitoleic acids [47]. While the **NpCN1** nitrile band is relatively weak in the PC loadings plots, there is a small positive contribution in PC-1 and a small negative contribution in PC-3 (Figure 5D). It is known that that the level of lipid unsaturation can be seen through the ratio of **v**(C=C)/**v**(CH_2_) [47]; here, the intensities of Raman bands were around 1657/1440 cm^−1^. In the PC-3 loadings plot, this high ratio relative to the spectra and other loadings shows that the PC-3 map was imaging TAGs with a higher degree of unsaturation. Furthermore, the negative contribution of 2232 cm^−1^ in PC-3 is correlated to the weaker contribution of the 1744 cm^−1^ peak, assigned with **v**(C=O) and assigned to mostly neutral lipids and TAGs. In PC-3, the bands at 1298 cm^−1^ and 1128 cm^−1^ are also negatively correlated; both bands are associated with neutral lipids and fatty acids. A combination of the results from PC-1 and PC-3 suggests that that **NpCN1** is correlated with polyunsaturated TAGs, and could be showing selectivity for particular classes of lipids in the lipid droplet core.

**Figure 6:**
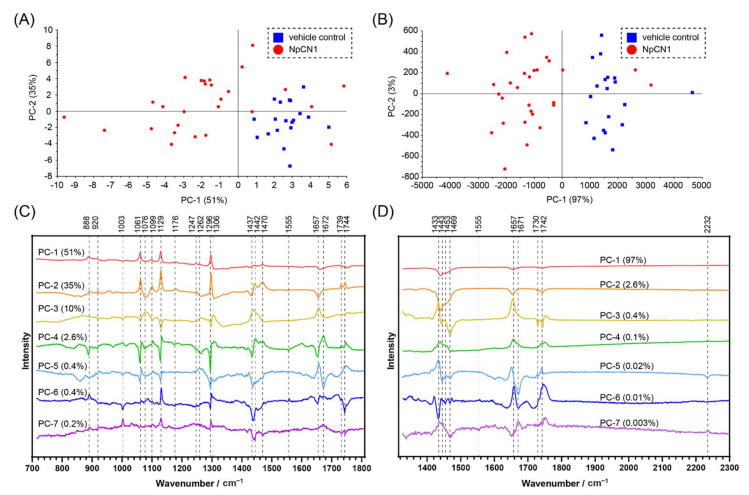
PCA of the average spectra of individual 3T3-L1 cells dosed with **NpCN1** (20 μM, 4 h) or equivalent volume DMSO as a vehicle control. Scores plots of the spectra from (**A**) 715 to 1806 cm^−1^ and (**B**) 1327 to 2304 cm^−1^ show a separation of the populations of cells. (**C**,**D**) show the loadings of the scores plotted in (**A**,**B**), respectively, along with the contribution of each PC.

The PCA on populations of cells provides further information about the interaction of **NpCN1** with cellular biomolecules. An average spectrum was obtained from the map of each 3T3-L1 adipocyte dosed with either **NpCN1** or an equivalent volume of DMSO and then compared using a PCA, producing scores plots with respective loadings plots (Figure 6). The PC-1 vs. PC-2 scores plot showed a distinct grouping of the two different cell populations for the spectra from 1327 to 2304 cm^−1^ (Figure 6B), which was expected as the peak from 2232 cm^−1^ should distinguish the populations. This general grouping was also observed in the PC-1 vs. PC-2 scores plot from 715 to 1806 cm^−1^, though it is not as specific. This shows that **NpCN1** can be used to distinguish dosed and control cells in a region of the spectrum without a specific Raman tag. The loadings provide further insight into the chemical composition of the cell and the localisation of **NpCN1**. Signals centred around 1440 cm^−1^, 1657 cm^−1^, and 1744 cm^−1^ appear as broad spectral bands in the extracted spectra (Figure 4); while broadly assigned to lipids, these bands are also known to overlap with various amide, protein, and nucleic acid bands [46]. It can be difficult to deconvolute and specifically assign broad spectral bands. However, the loadings from the PCA of the average spectra separated these broad bands into narrower and more distinct peaks that correspond to a sub-group of intracellular biomolecules that are most important in differentiating the cells treated with the probe versus the controls. This is most notable in the 1657 cm^−1^ peak; in both the PC-5 and PC-7 loadings, there is a peak around 1671 cm^−1^ not evident in the normal spectra that is correlated with the peak at 2232 cm^−1^, while a peak around 1657 cm^−1^ is negatively correlated against these peaks (Figure 6D). It is thus expected that the 1671 cm^−1^ peak is associated with lipids while the peak at 1657 cm^−1^ is more associated with the α-helix potein amide I peak that appears in the same general region. The use of **NpCN1** in combination with the various loadings plots thus helps overcome the issue of deconvoluting broad spectral bands and allows the assignment of lipid bands and how they change in response to the presence of the probe.

## 4. Discussion

Some organelles have a characteristic peak in vibrational spectroscopy; for instance, cytochrome c, a mitochondrial protein, exhibits a characteristic resonant Raman signal when excited with the 532 nm laser [48]. However, most organelles do not have unambiguous markers and spectral overlap can complicate the identification of organelles. In order to gain more biological information, it is, therefore, necessary to apply chemical tools to increase the specificity and selectivity for analytes or organelles of interest. Bioorthogonal Raman tags in the biological-silent region from 1800 to 2800 cm^−1^ have enabled the identification of organelles, though many approaches rely on resonance Raman (RR) [49], stimulated Raman scattering (SRS) [24], or surface-enhanced Raman spectroscopy (SERS) [50] to enhance the signal. **NpCN1** represents the first small molecule fluorescent probe incorporating a Raman tag for lipid droplets, and was imaged with both conventional Raman and fluorescent techniques. **NpCN1** also presents a new potential approach for investigating diseases associated with lipid metabolism such as diabetes, due to the affinity of **NpCN1** with certain classes of lipids, particularly polyunsaturated TAGs.

Though the identification of cellular lipids is possible with either fluorescent probes or label-free Raman spectroscopy alone, a bimodal approach with **NpCN1** allows for confirmation of localization across two techniques. Our studies provide a proof-of-concept that can be later applied to studying organelles that do not have well-established spectral bands for identification, such as lysosomes. While good Raman spectral results were obtained using **NpCN1**, the spectral signal of the nitrile peak was still somewhat weak, despite the addition of a benzene group to improve polarizability. A stronger signal may be obtained by attaching a moiety with better polarizability in future probes, such as a butadiyne tag [22]. However, the selective synthesis of unsymmetrical diynes remains challenging.

We have demonstrated that conventional Raman spectroscopy is possible with **NpCN1** through the careful selection of a fluorescent scaffold and excitation lasers for spectroscopy, overcoming potential issues with fluorescence background. The Raman mapping of the probe in cells resulted in minimal fluorescence background in the signal, comparable to the spectra obtained for the vehicle control cells (Figure S4). Following this approach, any small molecule dual fluorescence-Raman probe could be theoretically compatible with Raman spectroscopy, provided the Raman excitation laser does not overlap with the fluorophore’s excitation profile. This extends the classes of fluorophores available for use as scaffolds in future probes.

The spectral properties of naphthalimides such as **NpCN1** are compatible with the 785 nm laser commonly used for biological Raman studies, as this excitation provides a balance between scattering efficiency, minimisation of sample damage, and deeper cell penetration compared to lower wavelengths higher in energy [51]. We have recently shown that 4-amino-1,8-naphthalimides can be easily functionalized [52], allowing for moieties such as Raman tags and organelle targeting groups to be incorporated in a modular approach. We are currently working to extend our approach with **NpCN1** to other organelles. Furthermore, this modular approach allows for the incorporation of tags for other imaging modalities, extending the bimodal approach seen with **NpCN1**.

## 5. Conclusions

**NpCN1** is the first small molecule organic bimodal sensor for lipid droplets that is both fluorescent and Raman active in the biologically silent region of the spectrum. **NpCN1** has similar fluorescent staining properties to existing probes such as Nile Red in 3T3-L1 adipocytes, and Raman mapping confirmed lipid droplet staining in both modalities, reporting not only localisation but also chemical composition. A principal component analysis allowed the deconvolution of otherwise overlapping spectral bands, and the **NpCN1** signal was used to distinguish lipids in the assignment of these biomolecules. This multimodal approach can be readily used with a range of other fluorophores, allowing for a more detailed biochemical analysis of cellular and subcellular environments.

## Data Availability

Data is contained within the article or supplementary materials.

## Supplementary Materials

The following are available online at https://www.mdpi.com/article/10.3390/cells10071699/s1, Scheme S1: Synthesis of NpCN1; Scheme S2: Synthesis of alkyne-tagged naphthalimide variant; Figure S1: Raman spectrum of alkyne tagged variant 4, in the solid state, taken with the 50X and 785 nm laser excitation; Figure S2: Raw Raman spectrum of NpCN1, in the solid state, taken with the 50X and 785 nm laser excitation. Processed spectrum is seen in Figure 2D; Figure S3: Cell viability of NpCN1 in 3T3-L1 cells; Figure S4: Bimodal cell studies of a representative fixed 3T3-L1 cell dosed with an equivalent volume of DMSO to cells dosed with NpCN1; Figure S5: Principal component analysis of the Raman spectra of a 3T3-L1 cell dosed with an equivalent volume of DMSO to cells dosed with NpCN1; Figure S6: Confocal microscopy images of live 3T3-L1 cells.

## References

[1] Wu D., Sedgwick A.C., Gunnlaugsson T., Akkaya E.U., Yoon J., James T.D. (2017). Fluorescent chemosensors: The past, present and future. Chem. Soc. Rev..

[2] Matthäus C., Bird B., Miljković M., Chernenko T., Romeo M., Diem M. (2008). Chapter 10 Infrared and Raman Microscopy in Cell Biology. Methods Cell Biol..

[3] Carter E.A., Tam K.K., Armstrong R.S., Lay P.A. (2009). Vibrational spectroscopic mapping and imaging of tissues and cells. Biophys. Rev..

[4] Diem M., Mazur A., Lenau K., Schubert J., Bird B., Miljković M., Krafft C., Popp J. (2013). Molecular pathologyviaIR and Raman spectral imaging. J. Biophotonics.

[5] Diem M. (2015). Raman Microspectroscopy of Cells and Tissue in Medical Diagnostics. Modern Vibrational Spectroscopy and Micro-Spectroscopy.

[6] Kolanowski J.L., Liu F., New E.J. (2017). Fluorescent probes for the simultaneous detection of multiple analytes in biology. Chem. Soc. Rev..

[7] Zhao J., Chen J., Ma S., Liu Q., Huang L., Chen X., Lou K., Wang W. (2018). Recent developments in multimodality fluorescence imaging probes. Acta Pharm. Sin. B.

[8] Hackett M., Aitken J.B., El-Assaad F., McQuillan J.A., Carter E.A., Ball H.J., Tobin M.J., Paterson D., De Jonge M.D., Siegele R. (2015). Mechanisms of murine cerebral malaria: Multimodal imaging of altered cerebral metabolism and protein oxidation at hemorrhage sites. Sci. Adv..

[9] Bader C.A., Shandala T., Carter E.A., Ivask A., Guinan T., Hickey S.M., Werrett M.V., Wright P.J., Simpson P.V., Stagni S. (2016). A Molecular Probe for the Detection of Polar Lipids in Live Cells. PLoS ONE.

[10] Bader C.A., Carter E.A., Safitri A., Simpson P.V., Wright P., Stagni S., Massi M., Lay P.A., Brooks D.A., Plush S.E. (2016). Unprecedented staining of polar lipids by a luminescent rhenium complex revealed by FTIR microspectroscopy in adipocytes. Mol. BioSyst..

[11] Sorvina A., Bader C.A., Caporale C., Carter E.A., Johnson I.R.D., Parkinson-Lawrence E.J., Simpson P.V., Wright P.J., Stagni S., Lay P.A. (2018). Lipid profiles of prostate cancer cells. Oncotarget.

[12] Das N.K., Dai Y., Liu P., Hu C., Tong L., Chen X., Smith Z.J. (2017). Raman Plus X: Biomedical Applications of Multimodal Raman Spectroscopy. Sensors.

[13] Huang Z., Lui H., McLean D.I., Korbelik M., Zeng H. (2005). Raman Spectroscopy in Combination with Background Near-infrared Autofluorescence Enhances the In Vivo Assessment of Malignant Tissues. Photochem. Photobiol..

[14] Bocklitz T.W., Salah F., Vogler N., Heuke S., Chernavskaia O., Schmidt C., Waldner M.J., Greten F., Bräuer R., Schmitt M. (2016). Pseudo-HE images derived from CARS/TPEF/SHG multimodal imaging in combination with Raman-spectroscopy as a pathological screening tool. BMC Cancer.

[15] Wang H., Fu Y., Zickmund P., Shi R., Cheng J.-X. (2005). Coherent Anti-Stokes Raman Scattering Imaging of Axonal Myelin in Live Spinal Tissues. Biophys. J..

[16] van Manen H.-J., Kraan Y.M., Roos D., Otto C. (2005). Single-cell Raman and fluorescence microscopy reveal the association of lipid bodies with phagosomes in leukocytes. Proc. Natl. Acad. Sci. USA.

[17] Alvarez-Puebla R.A., Pazos-Perez N., Guerrini L. (2018). SERS-fluorescent encoded particles as dual-mode optical probes. Appl. Mater. Today.

[18] Navas-Moreno M., Mehrpouyan M., Chernenko T., Candas D., Fan M., Li J.J., Yan M., Chan J.W. (2017). Nanoparticles for live cell microscopy: A surface-enhanced Raman scattering perspective. Sci. Rep..

[19] Kim H.-M., Kim D.-M., Jeong C., Park S.Y., Cha M.G., Ha Y., Jang D., Kyeong S., Pham X.-H., Hahm E. (2018). Assembly of Plasmonic and Magnetic Nanoparticles with Fluorescent Silica Shell Layer for Tri-functional SERS-Magnetic-Fluorescence Probes and Its Bioapplications. Sci. Rep..

[20] Jeong S., Kim Y.-I., Kang H., Kim G., Cha M.G., Chang H., Jung K.O., Kim Y.-H., Jun B.-H., Hwang D.W. (2015). Fluorescence-Raman Dual Modal Endoscopic System for Multiplexed Molecular Diagnostics. Sci. Rep..

[21] Yang G., Zhang Q., Liang Y., Liu H., Qu L.-L., Li H. (2019). Fluorescence-SERS dual-signal probes for pH sensing in live cells. Colloids Surf. A Physicochem. Eng. Asp..

[22] Yamakoshi H., Dodo K., Palonpon A., Ando J., Fujita K., Kawata S., Sodeoka M. (2012). Alkyne-Tag Raman Imaging for Visualization of Mobile Small Molecules in Live Cells. J. Am. Chem. Soc..

[23] Yamakoshi H., Dodo K., Okada M., Ando J., Palonpon A., Fujita K., Kawata S., Sodeoka M. (2011). Imaging of EdU, an Alkyne-Tagged Cell Proliferation Probe, by Raman Microscopy. J. Am. Chem. Soc..

[24] Zhao Z., Shen Y., Hu F., Min W. (2017). Applications of vibrational tags in biological imaging by Raman microscopy. Analysis.

[25] Chen Z., Paley D.W., Wei L., Weisman A., Friesner R.A., Nuckolls C., Min W. (2014). Multicolor Live-Cell Chemical Imaging by Isotopically Edited Alkyne Vibrational Palette. J. Am. Chem. Soc..

[26] Li X., Jiang M., Lam J.W.Y., Tang B.Z., Qu J.Y. (2017). Mitochondrial Imaging with Combined Fluorescence and Stimulated Raman Scattering Microscopy Using a Probe of the Aggregation-Induced Emission Characteristic. J. Am. Chem. Soc..

[27] de Pablo J.G., Chisholm D.R., Steffen A., Nelson A.K., Mahler C., Marder T.B., Peyman S.A., Girkin J.M., Ambler C.A., Whiting A. (2018). Tandem fluorescence and Raman (fluoRaman) characterisation of a novel photosensitiser in colorectal cancer cell line SW480. Analysis.

[28] Olzmann J.A., Carvalho P. (2019). Dynamics and functions of lipid droplets. Nat. Rev. Mol. Cell Biol..

[29] Melo R.C.N., D’Avila H., Wan H.-C., Bozza P., Dvorak A.M., Weller P.F. (2011). Lipid Bodies in Inflammatory Cells. J. Histochem. Cytochem..

[30] Ruggles K.V., Turkish A., Sturley S.L. (2013). Making, Baking, and Breaking: The Synthesis, Storage, and Hydrolysis of Neutral Lipids. Annu. Rev. Nutr..

[31] Gao Q., Goodman J.M. (2015). The lipid droplet—A well-connected organelle. Front. Cell Dev. Biol..

[32] Schindelin J., Arganda-Carreras I., Frise E., Kaynig V., Longair M., Pietzsch T., Preibisch S., Rueden C., Saalfeld S., Schmid B. (2012). Fiji: An open-source platform for biological-image analysis. Nat. Methods.

[33] Carter E.A., Rayner B.S., McLeod A.I., Wu L.E., Marshall C.P., Levina A., Aitken J.B., Witting P.K., Lai B., Cai Z. (2010). Silicon nitride as a versatile growth substrate for microspectroscopic imaging and mapping of individual cells. Mol. BioSyst..

[34] Duke R.M., Veale E.B., Pfeffer F., Kruger P.E., Gunnlaugsson T. (2010). Colorimetric and fluorescent anion sensors: An overview of recent developments in the use of 1,8-naphthalimide-based chemosensors. Chem. Soc. Rev..

[35] Hearn K.N., Nalder T.D., Cox R.P., Maynard H.D., Bell T., Pfeffer F.M., Ashton T.D. (2017). Modular synthesis of 4-aminocarbonyl substituted 1,8-naphthalimides and application in single molecule fluorescence detection. Chem. Commun..

[36] Lizzul-Jurse A., Bailly L., Hubert-Roux M., Afonso C., Renard P.-Y., Sabot C. (2016). Readily functionalizable phosphonium-tagged fluorescent coumarins for enhanced detection of conjugates by mass spectrometry. Org. Biomol. Chem..

[37] Leslie K.G., Jacquemin D., New E.J., Jolliffe K.A. (2018). Expanding the Breadth of 4-Amino-1,8-naphthalimide Photophysical Properties through Substitution of the Naphthalimide Core. Chem. A Eur. J..

[38] Greenspan P., Mayer E.P., Fowler S.D. (1985). Nile red: A selective fluorescent stain for intracellular lipid droplets. J. Cell Biol..

[39] Rumin J., Bonnefond H., Saint-Jean B., Rouxel C., Sciandra A., Bernard O., Cadoret J.-P., Bougaran G. (2015). The use of fluorescent Nile red and BODIPY for lipid measurement in microalgae. Biotechnol. Biofuels.

[40] Klymchenko A.S. (2017). Solvatochromic and Fluorogenic Dyes as Environment-Sensitive Probes: Design and Biological Applications. Acc. Chem. Res..

[41] Liang X., Yue X., Dai Z., Kikuchi J.-I. (2011). Photoresponsive liposomal nanohybrid cerasomes. Chem. Commun..

[42] Li W., Zamani R., Gil P.R., Pelaz B., Ibáñez M., Cadavid D., Shavel A., Alvarez-Puebla R.A., Parak W.J., Arbiol J. (2013). CuTe Nanocrystals: Shape and Size Control, Plasmonic Properties, and Use as SERS Probes and Photothermal Agents. J. Am. Chem. Soc..

[43] Lin-Vien D., Colthup N.B., Fateley W.G., Grasselli J.G. (1991). The –C≢N and –N≢C Groups. The Handbook of Infrared and Raman Characteristic Frequencies of Organic Molecules.

[44] Digel M., Ehehalt R., Füllekrug J. (2010). Lipid droplets lighting up: Insights from live microscopy. FEBS Lett..

[45] Greenspan P., Fowler S.D. (1985). Spectrofluorometric studies of the lipid probe, nile red. J. Lipid Res..

[46] Talari A.C.S., Movasaghi Z., Rehman S., Rehman I.U. (2015). Raman Spectroscopy of Biological Tissues. Appl. Spectrosc. Rev..

[47] Czamara K., Majzner K., Pacia M., Kochan K., Kaczor A., Baranska M. (2015). Raman spectroscopy of lipids: A review. J. Raman Spectrosc..

[48] Okada M., Smith N.I., Palonpon A.F., Endo H., Kawata S., Sodeoka M., Fujita K. (2012). Label-free Raman observation of cytochrome c dynamics during apoptosis. Proc. Natl. Acad. Sci. USA.

[49] Kuzmin A., Pliss A., Lim C.-K., Heo J., Kim S., Rzhevskii A., Gu B., Yong K.-T., Wen S., Prasad P.N. (2016). Resonance Raman Probes for Organelle-Specific Labeling in Live Cells. Sci. Rep..

[50] Langer J., Jimenez de Aberasturi D., Aizpurua J., Alvarez-Puebla R.A., Auguié B., Baumberg J.J., Bazan G.C., Bell S.E.J., Boisen A., Brolo A.G. (2020). Present and Future of Surface Enhanced Raman Scattering. ACS Nano.

[51] Butler H.J., Ashton L., Bird B., Cinque G., Curtis K., Dorney J., Esmonde-White K., Fullwood N.J., Gardner B., Martin-Hirsch P.L. (2016). Using Raman spectroscopy to characterize biological materials. Nat. Protoc..

[52] Adair L.D., Trinh N., Vérité P.M., Jacquemin D., Jolliffe K.A., New E.J. (2020). Synthesis of Nitro-Aryl Functionalised 4-Amino-1,8-Naphthalimides and Their Evaluation as Fluorescent Hypoxia Sensors. Chem. A Eur. J..

