# Supplementary material for: A Bimodal Fluorescence-Raman Probe for Cellular Imaging
Source: Cells. 2021 Jul 5;10(7):1699. doi: 10.3390/cells10071699 (PMC8303253; doi:10.3390/cells10071699)
Supplement: Supplementary file 1 [file cells-10-01699-s001.zip › cells-1239325-supplementary.pdf]

Electronic supplementary material for

A bimodal fluorescence-Raman probe for cellular imaging

Jiarun Lin [1, 2], Marcus E. Graziotto [1], Peter A. Lay [1, 2, 3] * and Elizabeth J. New [1,2,4] *

1 School of Chemistry, The University of Sydney, NSW 2006, Australia; jiarun.lin@sydney.edu.au (J.L.); marcus.graziotto@sydney.edu.au (M.E.G.)

2 The University of Sydney Nano Institute (Sydney Nano), The University of Sydney, NSW 2006, Australia

3 Sydney Analytical, The University of Sydney, Sydney, NSW 2006, Australia

4 Australian Research Council Centre of Excellence for Innovations in Peptide and Protein Science, The University of Sydney, NSW 2006, Australia

* Correspondence: +61 2 9351 4269, peter.lay@sydney.edu.au (P.A.L.); + 61 2 9351 3329, elizabeth.new@sydney.edu.au (E.J.N.)

Cell viability assays

Cell viability was determined using the MTT assay, making use of the conversion of MTT (3-(4,5-dimethylthiazol-2-yl)-2,5- diphenyltetrazolium bromide) to an insoluble purple formazan product by the mitochondrial dehydrogenase of viable cells. The insoluble product can be quantified spectrophotometrically upon dissolution in a suitable solvent.

3T3-L1 preadipocyte cells were seeded in each well of flat-bottomed plastic 96-well plates (Corning) and differentiated as described in the methods. **NpCN1** solutions were added to quintuplicate wells to give final concentrations ranging from 0 – 100 μM. Following 24 h incubation, MTT (0.5 mg/mL) was added to each well, and the plates were incubated for an additional 4 h. The culture medium was removed, and DMSO (110 μL) was added. The plates were shaken for 10 seconds and the absorbance measured immediately at 600 nm in a PerkinElmer EnSpire Plate Reader (PerkinElmer, Inc.). Values were normalised to controls containing maintenance media only.

General synthesis and characterisation

Reactions were performed under a nitrogen atmosphere unless otherwise indicated. Anhydrous 1,4-dioxane was obtained from Sigma Aldrich. Peptide-grade *N,N*-dimethylformamide (DMF) was obtained from LabScan. All other reagents and solvents were used as supplied unless indicated. Reactions were monitored by silica gel thin-layer chromatography plates (Merck, TLC Silica gel 60 F_{254}). Column chromatography was performed on silica gel 60 (Merck, 0.040-0.063 nm) or using a Biotage Isolera One. Preparative thin layer chromagraphy was performed on silica gel 60 glass preparative plates (Yucheng Chemical (Shanghai) Co. Ltd, 1 mm thick, 20 × 20 cm).

All NMR spectra were obtained at 300 K on Bruker AVANCE III 400 or Bruker AVANCE III 500 spectrometers equipped with a 5 mm BBFO probe with z-gradients. Deuterated $CDCl_3$ were obtained from Cambridge Isotope Laboratories. All chemical shifts are reported in ppm and all coupling constants are reported in Hz. ^1H NMR spectra are calibrated to trace isotopic impurities of the solvent (δ = 7.26 ppm for $CDCl_3$). ^1H NMR data are reported as: chemical shift, multiplicity, coupling constant(s) (*J*) and relative integral. The multiplicities are reported as one or more of the following: s = singlet, d = doublet, t = triplet, q = quartet, quint = quintet, sext = sextet, m = multiplet, br = broad. $^{13}C\{^1H\}$ NMR spectra are calibrated to trace isotopic impurities of the solvent (δ = 77.16 ppm for $CDCl_3$). $^{13}C\{^1H\}$ NMR data is reported as chemical shift.

Low resolution ESI and APCI mass spectrometry was performed on a Bruker AmaZon SL ion trap mass spectrometer. For low resolution ESI, samples were injected via flow injection at 0.3 mL/min in methanol or acetonitrile into an Apollo II source with nitrogen drying gas at 180 ˚C. For low resolution APCI, samples were placed in a melting point tube and inserted into the Bruker Apollo II APCI source with an atmospheric solid analysis probe attachment added with vaporisation temperature 400 ˚C and corona current 4 μA. High resolution ESI mass spectrometry was performed on a Bruker solarix 2XR Fourier Transform Ion Cyclotron Resonance Mass Spectrometer. Samples were injected using the supplied syringe pump at 180 μL/h with nebuliser flow 1 L/min and drying gas 4L/min at 180 ˚C.

Detailed synthesis

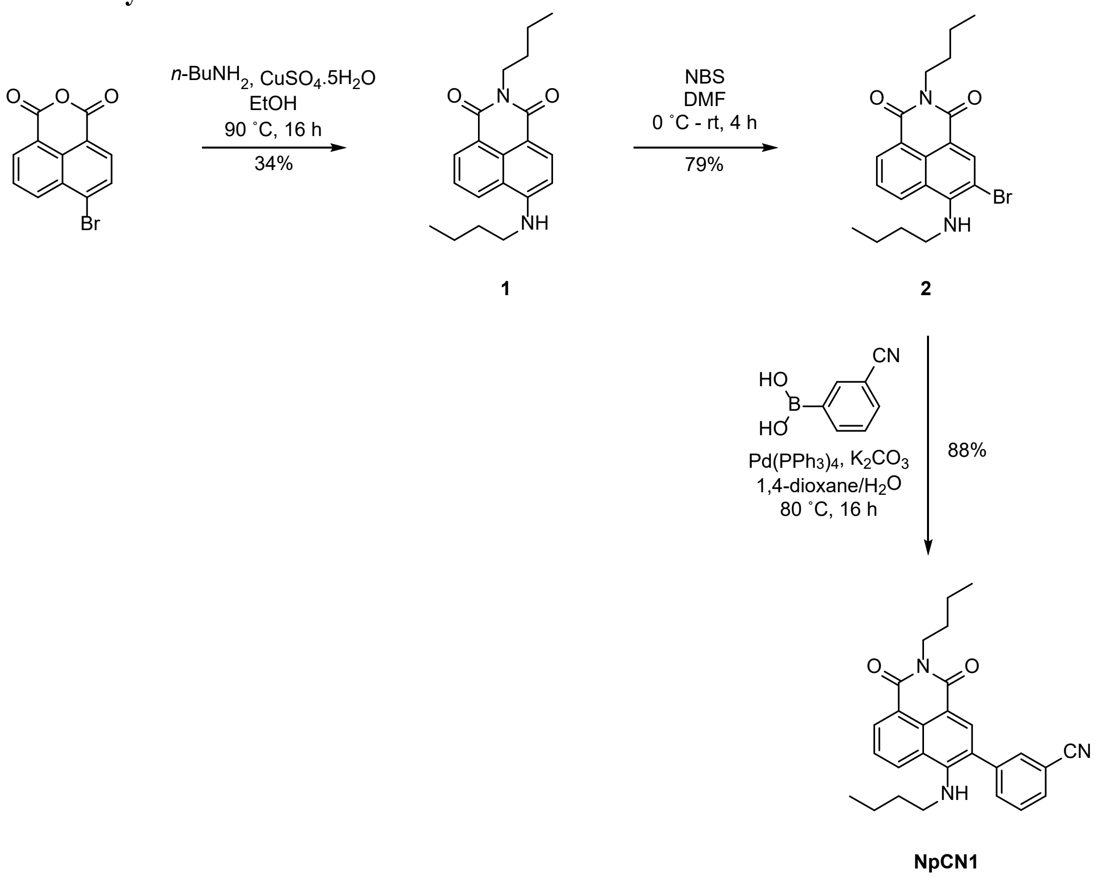

Scheme S1. Synthesis of **NpCN1**.

4-Butylamino-*N*-butyl-1,8-naphthalimide (**1**)

4-Bromonaphthalic anhydride (1.00 g, 3.60 mmol) and $CuSO_4{\cdot}5H_2O$ (0.090 g, 0.36 mmol) were suspended in absolute EtOH (30 mL). *n*-Butylamine (10 mL, 97 mmol) was added and the reaction was heated to reflux for 16 h. The reaction mixture was then poured into iced 1 M HCl (200 mL) and extracted with CH_2Cl_2 (3 × 100 mL). The organic extracts were washed with 0.1 M HCl (250 mL), dried over Na_2SO_4 and concentrated *in vacuo*. The crude was dissolved in EtOAc (30 mL) and filtered through a silica plug, and the solvent was removed *in vacuo.* The residue was recrystallised from absolute EtOH to afford **1** as gold needles (0.39 g, 34%).

^{1}H NMR (500 MHz, $CDCl_3$): δ 8.53 (dd, *J* = 7.3, 0.8 Hz, 1H), 8.42 (d, *J* = 8.4 Hz, 1H), 8.07 (dd, *J* = 8.4, 0.9 Hz, 1H), 7.55 (dd, *J* = 7.4, 0.8 Hz, 1H), 6.68 (d, J = 8.5 Hz, 1H), 4.15 (t, *J* = 7.6 Hz, 1H), 3.38 (t, *J* = 7.2 Hz, 1H), 1.81–1.75 (m, 2H), 1.73–1.67 (m, 2H), 1.55–1.48 (m, 2H), 1.46–1.39 (m, 2H), 1.01 (t, J = 7.3 Hz, 3H), 0.95 (t, *J* = 7.3 Hz, 3H).

^{13}C NMR (126 MHz, $CDCl_3$): δ 164.8, 164.2, 149.6, 134.5, 131.1, 129.9, 125.9, 124.7, 123.3, 120.3, 110.3, 104.4, 43.6, 40.1, 31.1, 30.4, 20.55, 20.46, 13.99, 13.95.

LRMS (APCI) m/z: $[M+H]^+$ Calcd for $C_{20}H_{25}N_2O_2^+$ 325; Found 325.

Characterisation data matches that reported in the literature.[37]

3-Bromo-4-butylamino-*N*-butyl-1,8-naphthalimide **(2)**

1 (500 mg, 1.5 mmol) was dissolved in DMF (7 mL) and cooled to 0 ˚C. A solution of freshly-recrystallised *N*-bromosuccinimide (0.37 g, 2.1 mmol) in DMF (10 mL) was added dropwise over 1 h. The reaction was warmed to rt and stirred for 3 h. The reaction mixture was diluted with 5% LiCl solution (100 mL) and extracted with EtOAc (50 mL). The organic extract was washed with 5% LiCl solution (5 × 50 mL), dried over $MgSO_4$ and concentrated *in vacuo*. Purification by column chromatography (SiO_2, 6% EtOAc in hexanes) afforded **2** (0.49 g, 79%) as a yellow powder.

^{1}H NMR (400 MHz, $CDCl_3$): δ 8.62 (s, 1H), 8.57 (d, J = 7.3 Hz, 1H), 8.45 (d, J = 8.6 Hz, 1H), 7.64 (t, J = 7.6 Hz, 1H), 4.15 (t, J = 7.6 Hz, 2H), 3.67 (t, J = 6.9 Hz, 2H), 1.78 – 1.64 (m, 4H), 1.53 – 1.37 (m, 4H), 0.99 – 0.94 (m, 6H).

^{13}C NMR (101 MHz, $CDCl_3$): 164.3, 163.2, 149.8, 135.6, 131.5, 130.7, 129.8, 125.3, 123.5, 123.4, 114.4, 110.0, 50.9, 40.3, 33.7, 30.6, 20.5, 20.1, 14.0, 13.9.

LRMS (APCI) *m/z*: $[M+H]^+$ Calcd for $C_{20}H_{23}BrN_2O_2^+$ 403 and 405; Found 403 and 405.

Characterisation data matches that reported in the literature.[37]

3-(3-Benzonitrile)-4-butylamino-*N*-butyl-1,8-naphthalimide (**NpCN1**)

2 (0.25 g, 0.62 mmol), 3-cyanophenylboronic acid (0.18 g, 1.2 mmol), K_2CO_3 (0.17 g, 1.2 mmol) and $Pd(PPh_3)_4$ (36 mg, 5 mol%) were placed under an N_2 atmosphere. A degassed 3:1 1,4-dioxane:H_2O (30 mL) mixture was added, and the reaction mixture was heated to 80˚C for 16 h. Upon completion, the reaction was diluted with H_2O (50 mL) and extracted with EtOAc (3 × 40 mL). The organic extracts were washed with brine (100 mL), dried over Na_2SO_4 and concentrated *in vacuo*. Purification by column chromatography (SiO_2, 25% EtOAc in hexanes) afforded **NpCN1** (0.23 g, 88%) as a yellow powder.

^{1}H NMR (500 MHz, $CDCl_3$): δ 8.61 (dd, J = 7.3, 0.9 Hz, 1H), 8.34 (dd, J = 8.5, 0.9 Hz, 1H), 8.32 (s, 1H), 7.80–7.79 (m, 1H), 7.75–7.70 (m, 3H), 7.65–7.61 (t, J = 7.7 Hz, 1H), 4.58 (t, J = 5.0 Hz, 1H), 4.17 (t, J = 7.6 Hz, 2H), 3.12 (q, J = 6.8 Hz, 2H), 1.73–1.67 (m, 2H), 1.50–1.40 (m, 4H), 1.26–1.18 (m, 2H), 0.96 (t, J = 7.4 Hz, 3H), 0.82 (t, J = 7.4 Hz, 3H).

^{13}C NMR (126 MHz, $CDCl_3$): δ 164.5, 164.0, 149.3, 141.1, 135.3, 133.9, 133.0, 131.6, 131.5, 130.1, 129.8, 128.8, 125.7, 123.6, 123.5, 123.4, 118.5, 113.6, 113.4, 49.8, 40.2, 33.2, 30.4, 20.5, 20.0, 14.0, 13.8.

HRMS (ESI) m/z: $[M+H]^+$ Calcd for $C_{27}H_{28}N_3O_2^+$ 426.2176; Found 426.2171.

^{1}H NMR of **NpCN1**

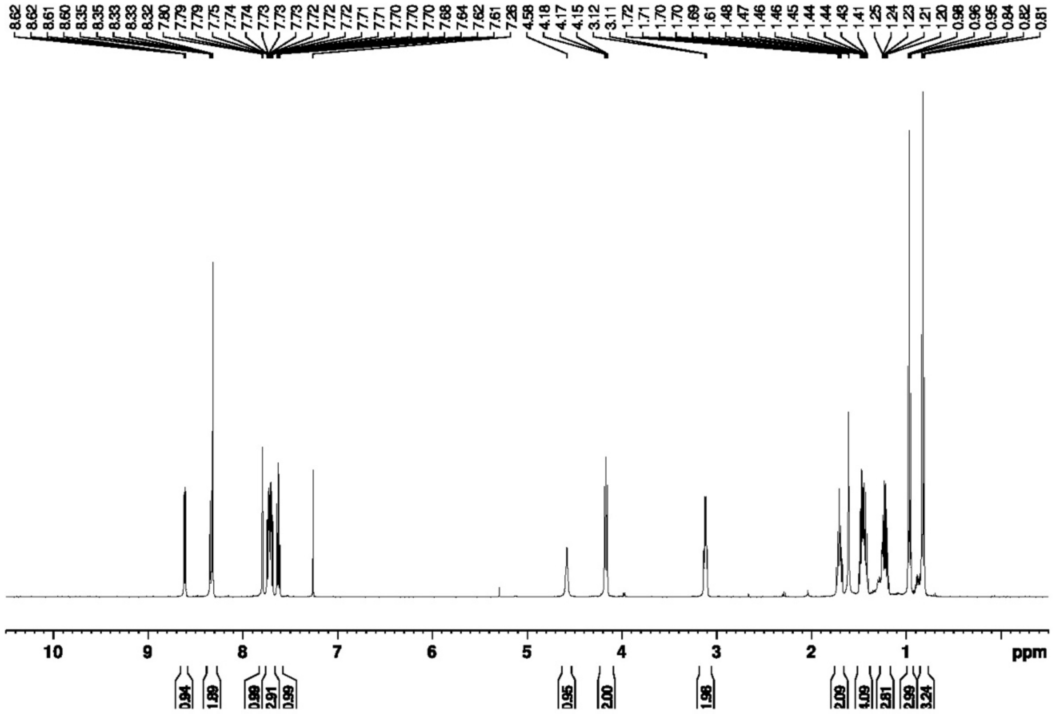

^{13}C NMR of **NpCN1**

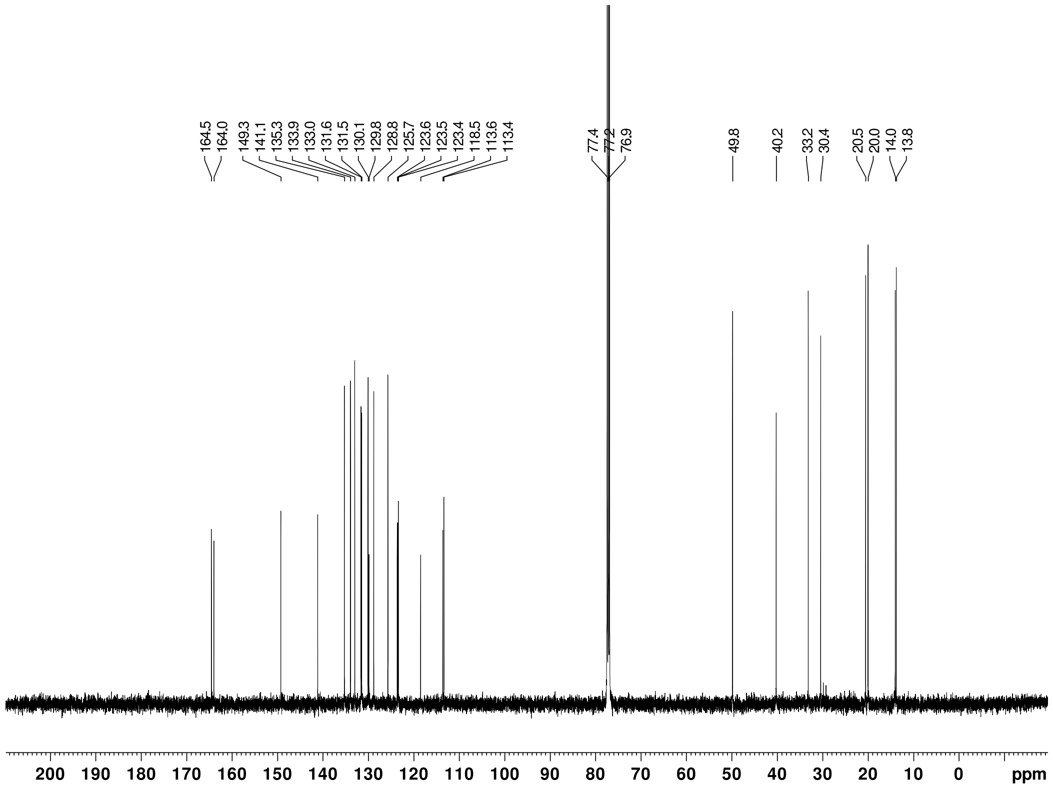

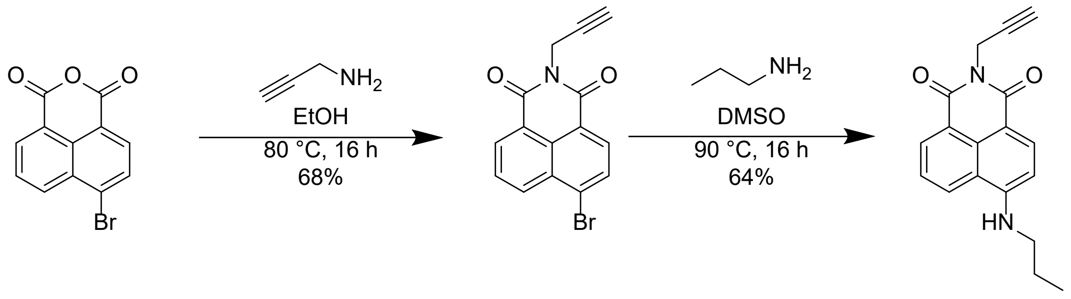

3 **4**

Scheme S2. Synthesis of alkyne-tagged naphthalimide variant.

6-bromo-2-(prop-2-yn-1-yl)-1*H*-benzo[*de*]isoquinoline-1,3(2*H*)-dione (3)

A solution of 4-bromo-1,8-naphthalic anhydride (3.01 g, 10.9 mmol) and propargylamine (750 µL, 11.7 mmol, 1.1 equiv) in EtOH (100 mL) was heated at reflux at 80 °C for 16 h under an N_2 atmosphere. The mixture was cooled to rt and then diluted using ice-cold H_2O (200 mL), with the resulting precipitate collected using vacuum filtration. Purification by column chromatography (SiO_2, 30% EtOAc in hexanes) afforded **3** (2.31 g, 68%) as a white powder.

^{1}H NMR (400 MHz, $CDCl_3$): δ 8.70 (dd, *J* = 7.3, 1.0 Hz, 1H), 8.59 (dd, *J* = 8.5 Hz, 1.0, 1H), 8.45 (d, *J* = 7.8 Hz, 1H), 8.06 (d, *J* = 7.9 Hz, 1H), 7.87 (dd, *J* = 8.5 Hz, 7.4, 1H), 4.96 (d, *J* = 2.5 Hz, 2H), 2.20 (t, *J* = 2.5 Hz, 1H).

^{13}C NMR (100 MHz, $CDCl_3$): δ 162.92, 162.90, 133.8, 132.5, 131.7, 131.3, 130.87, 130.86, 129.2, 128.3, 122.9, 122.0, 78.4, 70.8, 29.6.

LRMS (ESI) *m/z*: $[M+H]^+$ Calcd for $C_{15}H_9BrNO_2^+$ 314 and 316; Found 314 and 316.

Characterisation data matches that reported in the literature.[35]

2-(prop-2-yn-1-yl)-6-(propylamino)-1*H*-benzo[*de*]isoquinoline-1,3(2*H*)-dione (4)

A solution of **3** (102 mg, 0.324 mmol) and *n*-propylamine (120 µL, 1.46 mmol, 4.5 equiv) in DMSO (3 mL) was heated at 90 °C for 16 h under a N_2 atmosphere. The solution was cooled to rt and then diluted using ice-cold water (25 mL), with the resulting precipitate was collected using vacuum filtration. Purification by preparative thin layer chromatography (SiO_2, 30% EtOAc in hexanes) afforded **4** (61 mg, 64%) as a yellow powder.

^{1}H NMR (400 MHz, $CDCl_3$): δ 8.60 (dd, *J* = 7.3, 0.9 Hz, 1H), 8.48 (d, *J* = 8.4 Hz, 1H), 8.09 (dd, *J* = 8.5, 1.0 Hz, 1H), 7.61 (dd, *J* = 8.3, 7.4 Hz, 1H), 6.72 (d, *J* = 8.5 Hz, 1H), 5.31 (br, 1H), 4.94 (d, *J* = 2.95 Hz, 2H), 3.39 (t, *J* = 6.5 Hz, 2H), 2.17 (t, *J* = 2.4 Hz, 1H), 1.86 (sext, *J* = 7.3, 2H), 1.11 (t, *J* = 7.4, 3H).

^{13}C NMR (100 MHz, $CDCl_3$): δ 163.9, 163.2, 149.8, 134.9, 131.4, 129.9, 126.1, 124.7, 122.9, 120.2, 109.8, 104.4, 79.2, 70.0, 45.5, 29.2, 12.3, 11.6.

LRMS (ESI) *m/z*: $[M+H]^+$ Calcd for $C_{18}H_{17}N_2O_2^+$ 293; Found 293.

Characterisation data matches that reported in the literature.[36]

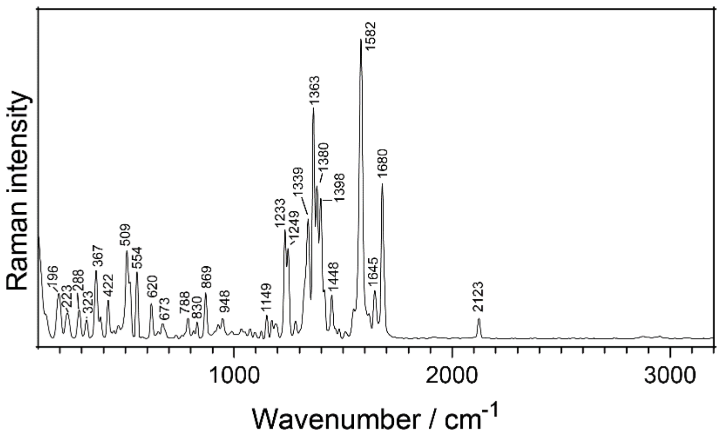

Figure S1. Raman spectrum of alkyne tagged variant **4**, in the solid state, taken with the 50X and 785 nm laser excitation.

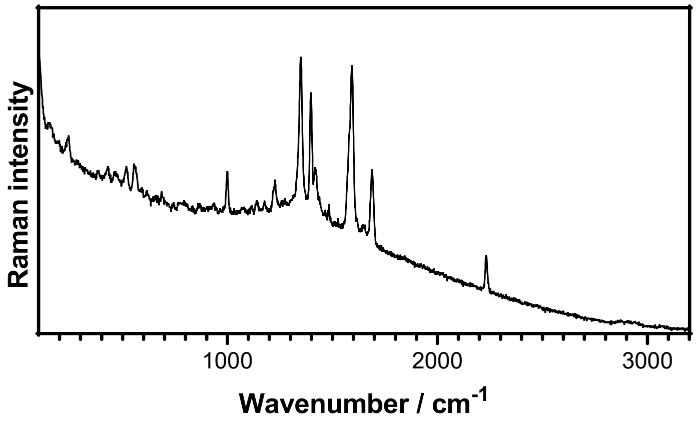

Figure S2. Raw Raman spectrum of **NpCN1**, in the solid state, taken with the 50X and 785 nm laser excitation. Processed spectrum is seen in Figure 2D.

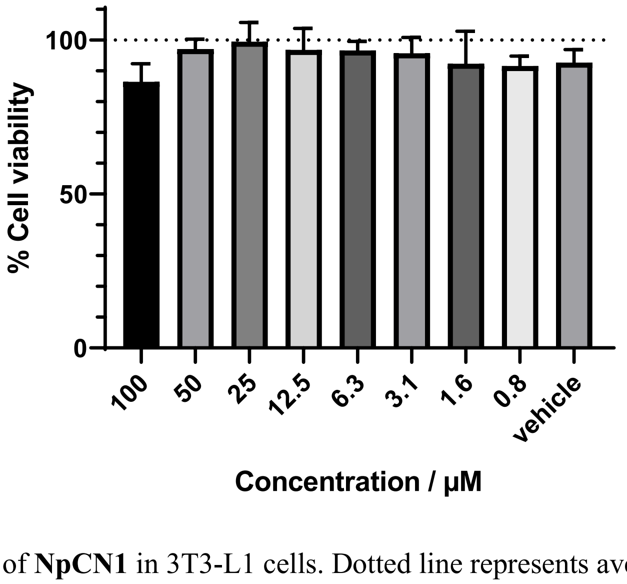

Figure S3. Cell viability of **NpCN1** in 3T3-L1 cells. Dotted line represents average cell viability of controls containing maintenance media only.

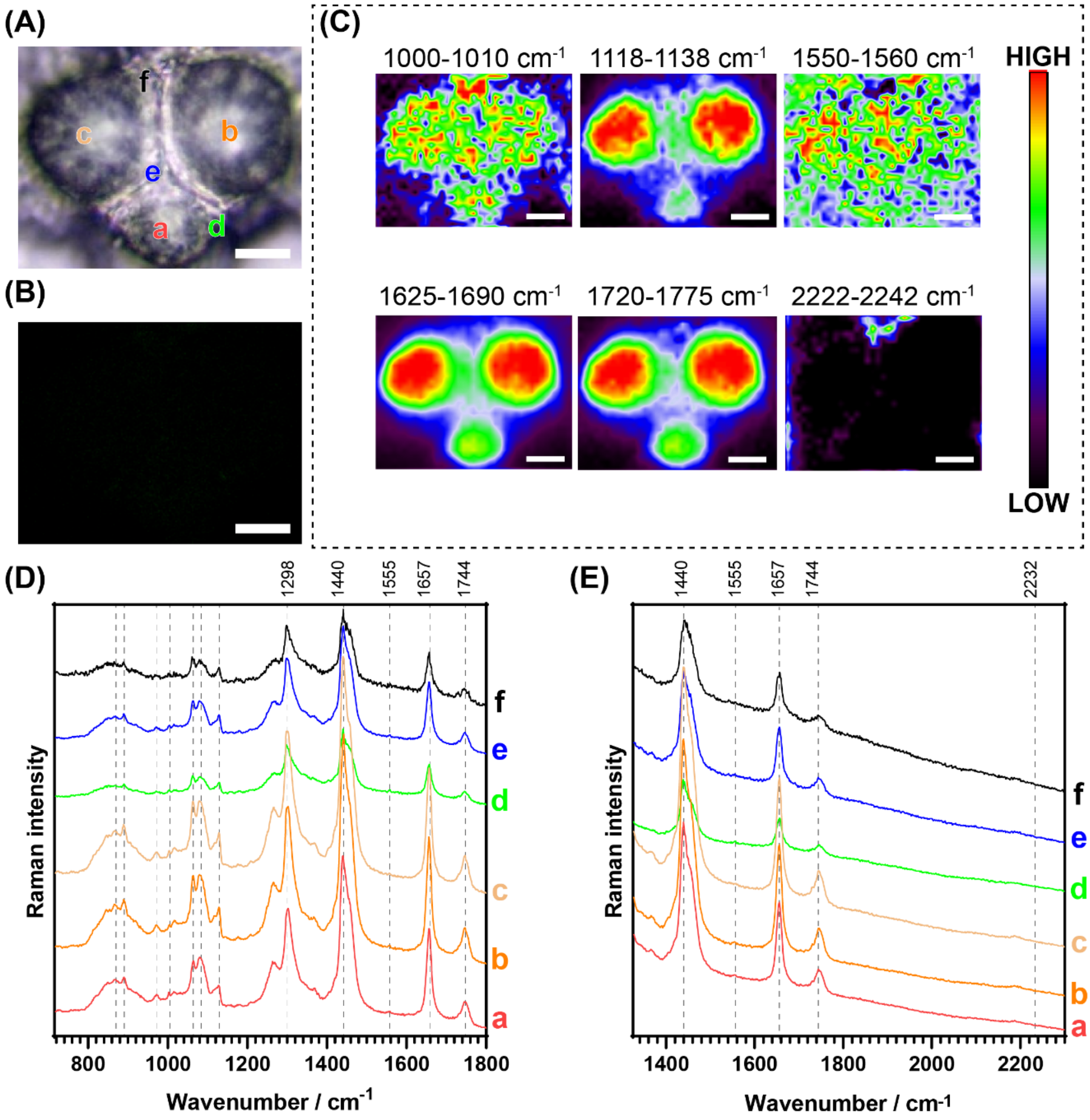

Figure S4. Bimodal cell studies of a representative fixed 3T3-L1 cell dosed with an equivalent volume of DMSO to cells dosed with **NpCN1**. (**A**) shows the bright field and (**B**) shows the confocal fluorescence image of the cell. (**C**) shows Raman maps with the distribution of phenylalanine (1000-1010 cm^{-1}), lipid C-C/fatty acids (1118-1138 cm^{-1}), tryptophan (1550-1560 cm^{-1}), lipid C=C (1625-1690 cm^{-1}), lipid esters (1720-1775 cm^{-1}) and **NpCN1** (2222-2242 cm^{-1}); maps obtained via calculation of the signal-to-baseline of spectra. (**D**) and (**E**) show selected spectra extracted from locations **a-f** as shown in (**A**). Scale bar represents 10 μm for all images and maps.

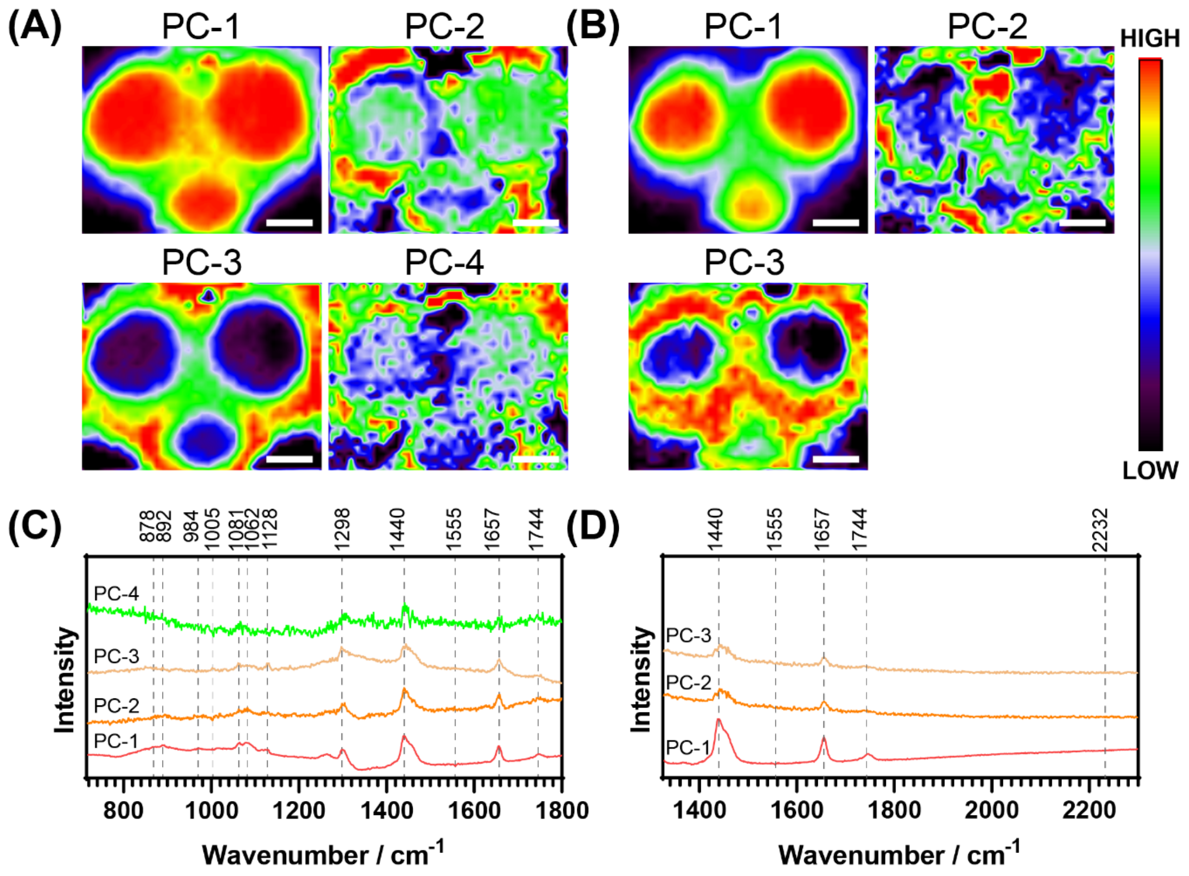

Figure S5. Principal component analysis of the Raman spectra of a 3T3-L1 cell dosed with an equivalent volume of DMSO to cells dosed with **NpCN1**; cell is the same representative cell shown in Figure S4. (**A**) and (**B**) show the PC maps of each of the scores of the four main PC of the spectra collected from 715-1806 cm^{-1} and 1327-2304 cm^{-1} respectively. **(C)** and **(D)** show the loadings of the scores mapped in (**A**) and (**B**) respectively. Scale bar represents 10 µm for all maps.

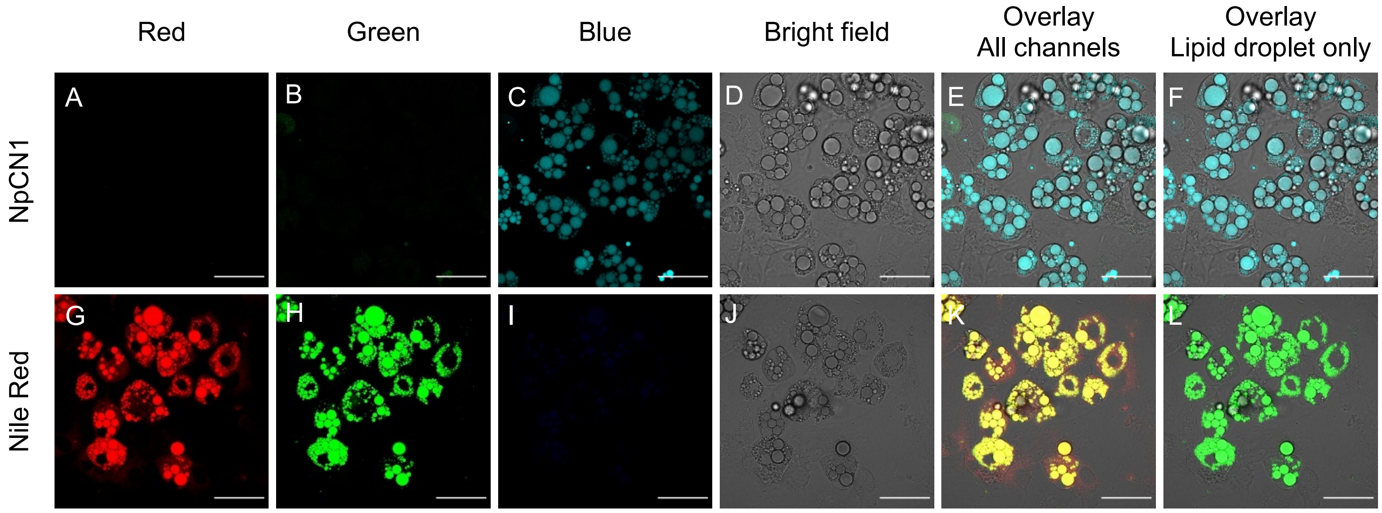

Figure S6. Confocal microscopy images of live 3T3-L1 cells treated with (**A-F**) **NpCN1** (20 µM, 40 min) or (**G-L**) Nile Red (10 µM, 40 min). Live cells were imaged in (**A,G**) red (λ_{ex} = 561 nm, λ_{em} = 570-670 nm), (**B,H**) green (λ_{ex} = 488 nm, λ_{em} = 500-600 nm) and (**C,I**) blue (λ_{ex} = 405 nm, λ_{em} = 450-550 nm) channels, along with (**D,J**) bright field images. (**E,K**) shows overlay of all channels; (**F**) shows overlay of C and D, (**L**) shows overlay of H and K. Scale bar on all images represents 50 µm.
